# Household-level data on food-water-energy nexus consumption in the urban areas of the Pune Metropolitan Region, India

**DOI:** 10.1016/j.dib.2026.112557

**Published:** 2026-02-07

**Authors:** Yuanzao Zhu, Christian Klassert, Vishal Gaikwad, Bernd Klauer, Erik Gawel

**Affiliations:** a Helmholtz Centre for Environmental Research - UFZ, Permoserstr. 15, 04318 Leipzig, Germany; b Gokhale Institute of Politics and Economics, Shivaji Nagar 846, 411004 Pune, Maharashtra, India; c Faculty of Economics and Business Management, Institute of Infrastructure and Resources Management, Leipzig University, Grimmaische Str. 12, 04109 Leipzig, Germany

**Keywords:** Socioeconomic survey, Intermittent water supply, Water demand, Energy demand, Utility bills, Household characteristics, Developing region

## Abstract

This article presents household-level socioeconomic data on food-water-energy nexus consumption collected through a survey conducted during the first quarter of 2020 in the urban areas of the Pune Metropolitan Region, India. The dataset includes 1872 observations from households residing in both formal and informal settlements. Data were collected via door-to-door interviews in the local language using a comprehensive, structured questionnaire administered through a computer-assisted web interviewing mobile application developed by the World Bank. Quality control was ensured through digital data capture, daily monitoring during fieldwork, and post-collection data validation. The dataset comprises 606 variables, including consumption data for water, energy, and food, alongside socioeconomic factors such as household composition, income, housing conditions, migration history, and household-level strategies to cope with intermittent water supply. The dataset can be used for econometric modeling of household demand, parameterization of multi-agent models, comparative analyses across regions, and empirical studies examining household challenges related to water, energy, and food security.

Specifications Table

**Table:** 

Subject	Social Sciences
Specific subject area	Socioeconomic survey on household water, energy, and food consumption
Type of data	Type of data: Table (.csv format) Supporting material (questionnaire) Data format: Cleaned Raw Data
Data collection	The data were collected through door-to-door interviews conducted in Marathi using a structured questionnaire digitized with the Survey Solutions software during the period from January 26 to March 23, 2020. The average interview duration was 42 min. A stratified random sampling approach was used to select households from four categories based on their housing conditions in the urban agglomeration of Pune and Pimpri-Chinchwad in the Pune Metropolitan Region.
Data source location	Country: India State: Maharashtra City/Town/Region: The Municipal Corporation Areas of Pune and Pimpri-Chinchwad
Data accessibility	Repository name: GESIS Data identification number: https://doi.org/10.7802/2867 Direct URL to data: https://search.gesis.org/research_data/SDN-10.7802–2867
Related research article	Y. Zhu, E. Gawel, B. Klauer, & C. Klassert, Impacts of Intermittent Water Supply on Household Electricity Demand: An Econometric Analysis for the Pune Metropolitan Region, India. Water Resour. Econ. (2024), 100,250. https://doi.org/10.1016/j.wre.2024.100250

## 1 Value of the Data

• The dataset provides comprehensive household-level water, energy, and food consumption and socioeconomic data for the urban areas of a major metropolitan region in India. As the first dataset of this scope for this region and among limited comparable nexus-integrated datasets available in developing regions, it fills a critical gap in the literature.
• The dataset includes precise consumption data for piped water and electricity sourced directly from utility bills. These variables are valuable for estimating demand functions using econometric models.
• The dataset can be used for empirical studies to better understand household challenges and behaviors related to water, energy, and food consumption, in particular, the strategies households employ to cope with an intermittent water supply.
• The dataset can be used for targeted analyses of specific aspects of the food-water-energy nexus at the household level, or for parameterizing households in large mathematical models, such as multi-agent models, for analyzing policy implications with regards to nexus issues.
• The dataset can be used for comparative analyses with similar information from other regions, supporting broader research and policy development.

## 2 Background

Both empirical and conceptual studies have demonstrated the close interconnections of household consumption of food, water, and energy [[1], [2], [3], [4], [5], [6], [7], [8], [9]], particularly in regions with intermittent water supplies [7], affecting over one billion people globally [10,11]. However, comprehensive household-level data on food, water, and energy consumption patterns and related socioeconomic factors is lacking in developing regions. Therefore, we conducted an in-depth socioeconomic household survey focusing on the food-water-energy nexus in urban areas of the Pune Metropolitan Region, one of India’s largest metropolitan regions, where rapid urbanization and population growth have exacerbated challenges related to the nexus which are expected to intensify in the future [12,13].

This data article is associated with a dataset [14] published in the GESIS repository and complements an original research article [8] analyzing the impact of intermittent water supplies on household electricity demand. This article provides a detailed account of the survey design and methods, expanding on the limited description of the data collection method presented in the research paper, where only a small fraction of the data was used for analysis. Here, we document and instruct the use of the full dataset to help other researchers understand and use the data for future studies.

## 3 Data Description

**Fig. 1:**
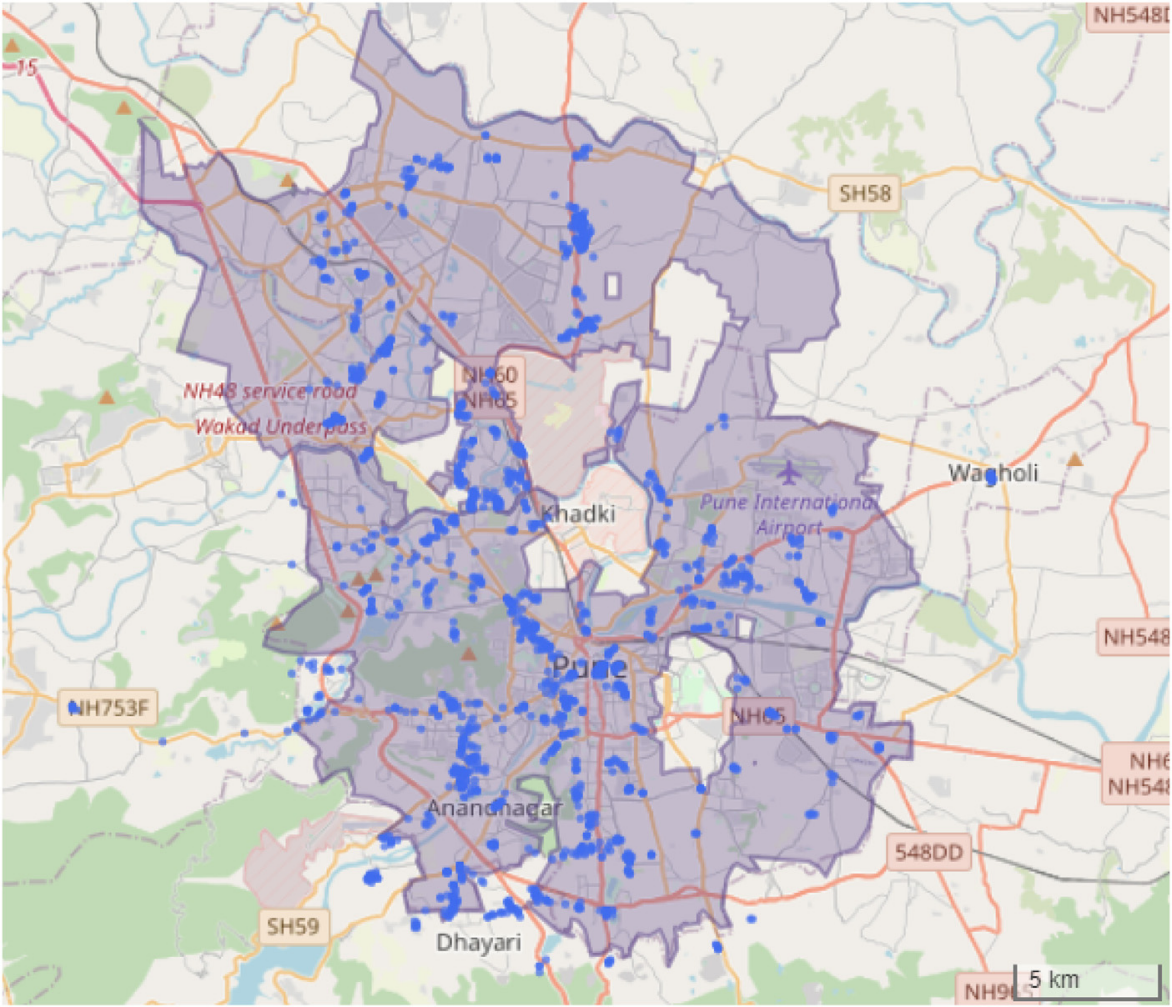
Spatial distribution of the 1872 survey observations within the administrative boundaries of the urban agglomeration of Pune and Pimpri-Chinchwad (excluding the cantonment areas), as mapped using OpenStreetMap.

The dataset includes 1872 valid household observations collected through interviews conducted between January 26 and March 23, 2020, in the urban agglomeration of Pune and its neighboring city Pimpri-Chinchwad, both of which are located in the Pune Metropolitan Region (see Fig. 1).

The dataset is provided as a single comma-separated values (.csv) file, which can be accessed using Microsoft Excel, R Studio, and other statistical software. The data file is accompanied by the questionnaire file in PDF format. Both files are publicly available in the GESIS repository [14]. The questionnaire file includes all survey questions, response options, and branching logic used during the structured interviews, as well as the corresponding variable code(s) for each survey item. Further details on the structure of the questionnaire are provided in the Section 4.1.

The data file contains cleaned raw data with 606 columns, each representing a specific variable identified by the unique variable code displayed in the top row. Users can refer to these codes in the accompanying questionnaire file, which provides detailed information about the survey question, available options, and any relevant conditional logic linked to the variable. Notably, the first column, labeled “*interview__key*”, serves as a unique identifier for each household observation. This allows users to match and merge different subsets of the data during analysis.

At the end of the data table, columns 577 to 606 contain verified information on household piped water and electricity consumption, obtained directly from utility bills. For piped water, the dataset includes information extracted from a maximum of one water bill per observation. Each bill details the total water consumption for the household over a specific period, with the bill amount representing the payment required for that usage. The recorded variables are: the water bill amount in Indian Rupees (“*waterbill_amount_1*”), the quantity of water consumed in cubic meters (“*waterbill_consumption_1*”), the date on which the bill was issued in month/day/year format (“*billing_end*”), and the number of days covered by each bill (“*billing_period*”). Regarding household electricity consumption, data from multiple months are available and stored under variables labeled by the corresponding month in the format of “*Month_Year*”. These variables capture household electricity consumption in kilowatt-hours per month, covering the period from January 2018 to February 2020, when data was available. For these verified consumption variables, *NA* values indicate either that the household did not provide utility bills, the bill photo was of insufficient quality for extraction, or bills were outdated (e.g., bills for 2014). Further details on how these variables are identified are provided in the Section 4.5.

In addition to the consumption variables, the dataset also includes variables capturing external infrastructure and socioeconomic factors, such as water supply infrastructure characteristics (municipal supply reliability measured in hours per day, supply frequency, metered vs. unmetered status, gap between supply periods), electricity supply reliability (hours per day, interruption patterns, backup generator ownership), and access to alternative water sources (tanker water, wells/borewells, bottled water, public stand posts, etc.). It also includes household coping strategies reflecting infrastructure constraints, such as water storage capacity, water pump ownership and operation patterns, and prioritized solutions during supply shortages. Socioeconomic variables are also included, such as household composition (size, age structure), income level and sources, housing conditions (ownership, housing type, number of rooms), education level of the household head, and migration history.

It should be noted that not all responses to all questions from the full questionnaire are included in the published data file. Although the survey was conducted anonymously and no personal information was collected, additional steps were taken to ensure complete respondent anonymity and full privacy protection. Variables from some open-ended questions, as well as any variables that could potentially lead to the identification of the neighborhood of the interviewed households (e.g., GIS coordinates of the nearest street corner), have been removed from the published data file. Researchers may contact the author to request access to these excluded variables for justified analyses in the form of secondary data.

No sampling weights were applied, as the survey sample is considered representative of the entire urban area of the Pune Metropolitan Region, excluding cantonment areas. Details on the sampling method are provided in the Section 4.2.

## 4 Experimental Design, Materials and Methods

### 4.1 Questionnaire design

The survey questionnaire was developed to capture the complex and diverse food-water-energy nexus consumption patterns in the study region. It contains over 300 questions to address this complexity; many of these questions are conditional, meaning whether or not a household needs to answer them depends on their responses to previous questions.

The structure of the questionnaire and the formulation of the questions were based on established instruments in India, particularly, the Household Consumption Expenditure Surveys conducted by the Indian National Sample Survey Office (NSSO surveys) [15,16] and the India Human Development Surveys (IHDS surveys) [17,18]. To allow for comparison and ensure compatibility with existing household-level datasets, definitions of key socioeconomic variables, such as household composition and income category, were adopted from the NSSO and IHDS questionnaires. For example, we adopted the IHDS definition of a household as all individuals living under the same roof and sharing a kitchen for at least six months, as well as the definitions of age groups for children, teenagers, adults, and seniors. This standardization enables researchers to link this dataset with national household surveys and external data sources that use comparable variable definitions, facilitating integration for comparative or complementary analyses.

The questionnaire is structured into eight sections, as detailed in the accompanying questionnaire file. The first and last sections are to be completed by the enumerator, respectively, to record the general housing type and area observed before the interview and to provide an overall assessment of the interview and any additional remarks from the enumerator after the interview. The six main sections, which are to be completed with information obtained during the interview, cover the following topics: (i) household characteristics, (ii) water, (iii) energy, (iv) housing conditions, income, and food, (v) migration, and (vi) closing questions with open-ended items for additional remarks from the surveyed household.

The questionnaire was initially drafted as a Word document and then digitized using the Survey Solutions software [19] to facilitate administration and quality control.

### 4.2 Survey sampling

The survey focused on the urban portions of the Pune Metropolitan Region, including the Pune City and Haveli sub-districts (excluding cantonment areas). We classified households into four categories based on expected differences in water use: (1) standard urban households; (2) households in informal settlements, where access to water is often restricted; (3) households in housing societies or townships, where water use is often managed collectively at the building level; and (4) peri‑urban or rural households at the urban boundaries, where water supply conditions might be complex and multiple water sources are likely to be used. We applied a stratified sampling formula [20] to determine the sample size:

$$n={\left[\frac{1}{N}+\frac{N-1}{N}\frac{1}{PQ}{\left(\frac{k}{z}\right)}^{2}\right]}^{-1}$$

where N refers to the number of households, P is the proportion of households within the population and Q denotes the complement of P (i.e., *Q* = 1 - P), k is the desired level of precision, and z is the value of the normal standard coordinate for the desired level of confidence.

We used demographic data from the 2011 Census of India [21] at the city and village levels to determine the number of households in the surveyed area, and from the Pune Slum Atlas [22] for households living in informal settlements. To distinguish households residing in housing societies or townships from standard urban households, we assumed households living in dwellings with three or more rooms reside in guarded housing societies or townships. We used the Houselisting and Housing Primary Census Abstract data from the census [21] to determine referenced proportions. For the first three household categories, we further disaggregated the numbers for Pune and Pimpri-Chinchwad based on census data.

We calculated the sample size at a 95 % confidence level and 4 % precision. To ensure a spatially balanced representation of the surveyed area, we proportionally allocated the survey sample according to the population composition of different areas in Pune, Pimpri-Chinchwad, and surrounding areas.

During fieldwork, we employed a targeted sampling strategy with replacement: enumerators systematically approached households in each area until achieving the target sample size for that stratum. Specifically, enumerators were instructed to ask every second household on one side of the street for interviews; if rejected, they would approach the next household. When moving to the next street, the street side was rotated to ensure balanced spatial coverage. Only observations meeting a minimum data quality threshold of >80 % response rate (based on applicable, non-skipped questions) were accepted as valid observations in the final dataset. This systematic approach ensured the achieved sample composition matched our stratified targets, confirming population representativeness without requiring post-hoc weighting adjustments.

### 4.3 Pilot testing

We carried out the survey, including the pilot testing, in collaboration with the Gokhale Institute of Politics and Economics, a highly reputed and widely recognized research and training institute in Pune. We conducted a preliminary survey of 120 households from May 30 to June 9, 2019 across the cities of Pune and Pimpri-Chinchwad. The pilot survey used a paper-based version of the questionnaire, and the data collected from the interviews were manually entered into an Excel document.

Lessons learned from the pilot survey led to improvements in the questionnaire and survey design. Examples of key modifications include the following. First, we refined measurement units for water consumption based on household comprehension. During the pilot phase, we asked households for their perceived water consumption using the standard metric unit “liter/month” (e.g., “How much piped water do you typically use per month?”). However, this approach yielded uncertain and inconsistent responses, as most household respondents struggled to mentally aggregate daily water-use behaviors into monthly totals. Pilot testing revealed that households in the surveyed region were substantially more comfortable estimating their piped water and bottled water consumption when asked in “liter/day” units, as this time frame aligned with their daily water-use routines. We consequently modified the final questionnaire to use “liter/day” for these questions. Second, we incorporated locally-familiar measurement units, such as “bucket” (locally understood as approximately 20 liters), which enabled households to report consumption more intuitively. Third, we restructured questions with problematic framing. For instance, during the pilot phase, questions on water-related vulnerabilities used open-ended formats with academic language (e.g., “Have you felt vulnerable regarding water use during the past twelve months?”), which proved difficult for households to understand and generated inconsistent and redundant responses. We revised these to use simpler, more accessible framing (e.g., “Have you felt water use in your household problematic during the past twelve months?”) followed by structured multi-select response options (e.g., price affordability, water quality concerns, quantity insufficiency, supply interruptions) to improve comprehension, response consistency, and analytical utility. Beyond these examples, multiple other refinements were made based on pilot findings to address diverse household contexts and improve interview flow. Regarding survey design, in particular, we addressed inefficiencies arising from the paper-based pilot process by digitizing the questionnaire with the Survey Solutions software [19], enabling built-in validation and real-time data quality monitoring during the main survey phase.

### 4.4 Data collection

The main survey phase commenced in January 2020. We selected students majoring in economics from the local colleges to work as enumerators. In the week leading up to the survey, we conducted training workshops with these students, including a short pilot testing phase to make final improvements to the questionnaire and interview structure. This also allowed the students to understand the questionnaire and logics behind the questions, and to familiarize themselves with the interview procedure.

The survey was conducted via door-to-door interviews in Marathi based on the structured questionnaire in the Survey Solutions mobile app [19]. All interviewed households were randomly selected, and the household category was identified based on building appearances prior to the interview. Interviews were conducted with whichever adult household member was available and willing to participate at the time of contact. We did not intentionally pre-select which specific household member should respond to allow flexibility and increase participation rates. To document respondent characteristics, we recorded the type of respondent (with the variable “*respondent*”) at the end of each interview, distinguishing between male/female household head, and male/female household member. This allows researchers to examine whether respondent type affects data patterns.

To ensure that key information was captured, essential questions on water and energy consumption as well as certain socioeconomic factors were prioritized at the beginning of the interviews. In particular, to collect reliable consumption data, we asked households to show their recent piped water and electricity bills during the interviews. When interviewed households agreed to share their bills, we recorded the numbers from the bills as answers to the corresponding consumption questions. With permission, we photographed the consumption sections using the Survey Solutions mobile app. Care was taken to exclude any private information from the photos. These images were automatically uploaded to the Survey Solutions cloud server upon completion of the interview. The information from these photos was then used to identify variables regarding precise household consumption of piped water and electricity, as described in Section 3 and further explained in Section 4.5.

At the end of each interview, enumerators were asked to provide a self-evaluation of overall interview quality and additional remarks (i.e., the questionnaire section “Enumerator Part: Ending”). No monetary or material incentives were offered to households; participation was entirely voluntary based on household willingness to contribute to research.

### 4.5 Data cleaning

Upon completion of each interview, data were uploaded to the Survey Solutions cloud server via the mobile app [19]. The cloud server was available until the end of October 2020, and all data was deleted from the server afterwards. We conducted daily checks to identify and exclude incomplete or low-quality interviews. As explained in Section 4.2, only observations meeting a minimum threshold of response rate were accepted, we instructed enumerators to conduct additional interviews in the sampled area to meet targeted sample sizes when interviews were rejected due to incompleteness. After data collection, we conducted a thorough review of the data file, including cross-validation of responses and clarification with enumerators where necessary.

**Fig. 2:**
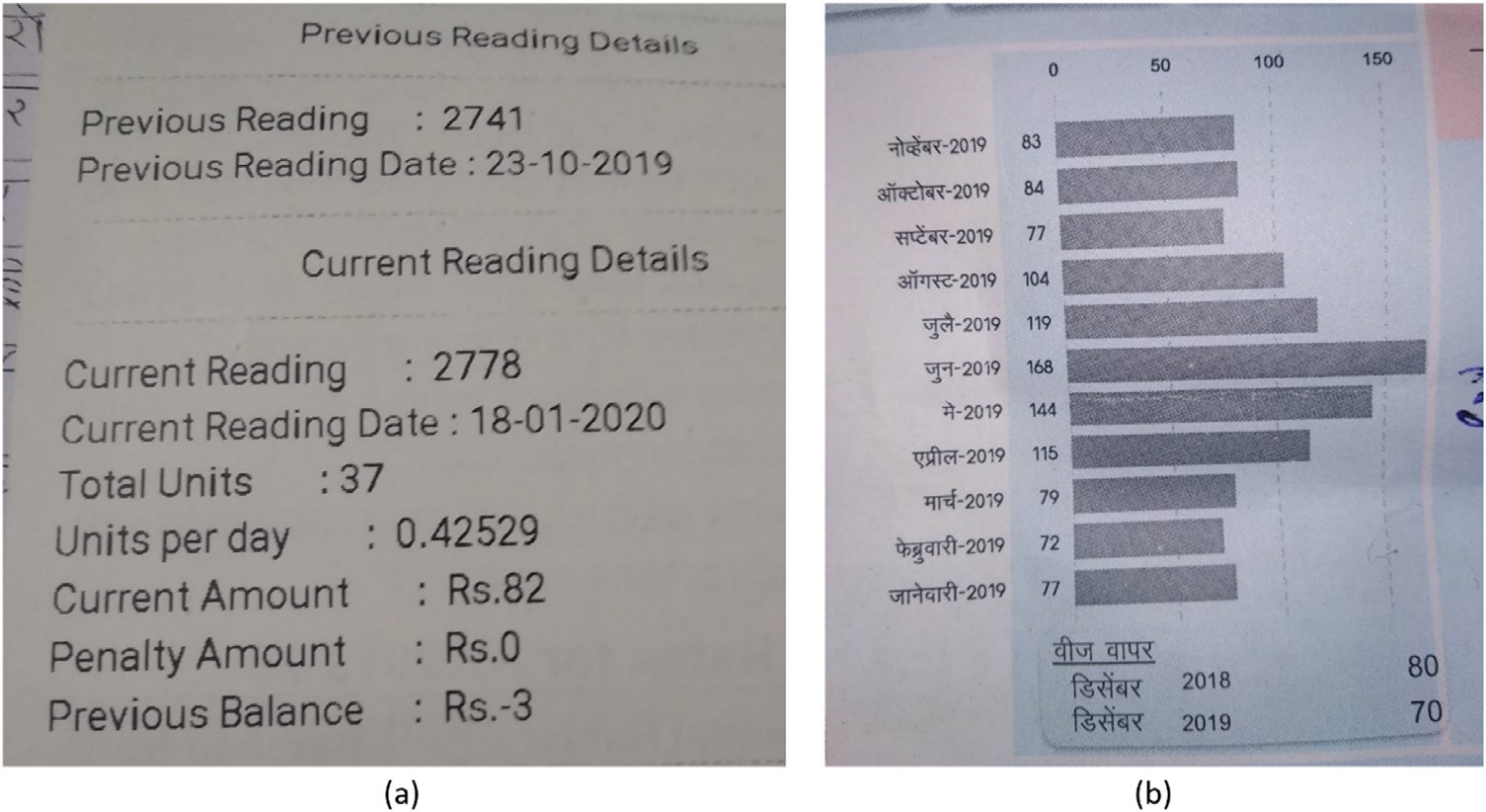
Example of utility bills recorded during the survey, with (a) a piped water bill that contains information on household piped water consumption and bill amount during a specified period, and (b) an electricity bill that contains information on household monthly electricity consumption over 13 months.

During data cleaning, we translated open-ended responses from Marathi to English where necessary. For utility bill data, we reviewed all uploaded photos and manually extracted the consumption information from photos of acceptable quality. Photos that were vague or incompletely captured were not used for extraction. The extracted information was then merged into the main data file using the unique household identifier (“*interview__key*”). Fig. 2 shows an example of the bill photos. The piped water bill typically contained data for a single period, while electricity bills included up to 13 months of consumption data, depending on the quality and completeness of the recorded photos. All missing values were coded as *NA*. These values result from two sources: the conditional skip logic, where questions did not apply to respondents based on prior answers, and the item non-response, where respondents chose not to answer applicable questions.

## Limitations

We aimed to create a comprehensive dataset covering a wide range of household information. However, we could not achieve the desired level of comprehensiveness for all 1872 survey observations, because data collection depended fully on households’ willingness to participate and provide the requested information. Some households did not have the time or patience to answer every question. We took measures to mitigate this issue, e.g., by prioritizing essential questions at the beginning of the interviews to ensure the collection of key information in cases where some households were unable to complete the entire interview. However, certain sections, such as the food section, which appeared later in the questionnaire and required detailed recall, may contain incomplete or less detailed responses. Furthermore, precise consumption data for piped water and electricity were only available from households able to access and willing to share recent utility bills during the interview. In some cases, bill information could not be used due to malfunctioning meters, or outdated or incomplete information.

Future studies may address these limitations by conducting in-depth interviews with fewer households, conducting interviews over time, or collaborating with utility providers. However, these approaches may compromise sample size, survey comprehensiveness, or respondent anonymity.

## Ethics Statement

The data were collected with the consent of the households interviewed. The authors have read and followed the ethical requirements for publication in Data in Brief, confirming that the current work does not involve human subjects, animal experiments, or data collected from social media platforms.

## CRediT Author Statement

**Yuanzao Zhu:** Conceptualization, Methodology, Formal Analysis, Investigation, Data Curation, Project Administration, Visualization, Writing- Original Draft, Writing- Review & Editing. **Christian Klassert:** Conceptualization, Methodology, Writing- Review & Editing, Supervision. **Vishal Gaikwad**: Investigation, Writing- Review & Editing. **Bernd Klauer:** Writing- Review & Editing, Supervision, Funding acquisition. **Erik Gawel:** Writing- Review & Editing, Supervision.

## Data Availability

GESISPune Household Food-Water-Energy Nexus Consumption Survey (Original data).
